# A novel organotypic 3D sweat gland model with physiological functionality

**DOI:** 10.1371/journal.pone.0182752

**Published:** 2017-08-10

**Authors:** Patricia Klaka, Sabine Grüdl, Bernhard Banowski, Melanie Giesen, Andrea Sättler, Peter Proksch, Thomas Welss, Thomas Förster

**Affiliations:** 1 Henkel AG & Co. KGaA, Düsseldorf, Germany; 2 Institute of Pharmaceutical Biology and Biotechnology, Heinrich-Heine-University, Düsseldorf, Germany; University of Alabama at Birmingham, UNITED STATES

## Abstract

Dysregulated human eccrine sweat glands can negatively impact the quality-of-life of people suffering from disorders like hyperhidrosis. Inability of sweating can even result in serious health effects in humans affected by anhidrosis. The underlying mechanisms must be elucidated and a reliable *in vitro* test system for drug screening must be developed. Here we describe a novel organotypic three-dimensional (3D) sweat gland model made of primary human eccrine sweat gland cells. Initial experiments revealed that eccrine sweat gland cells in a two-dimensional (2D) culture lose typical physiological markers. To resemble the *in vivo* situation as close as possible, we applied the hanging drop cultivation technology regaining most of the markers when cultured in its natural spherical environment. To compare the organotypic 3D sweat gland model versus human sweat glands *in vivo*, we compared markers relevant for the eccrine sweat gland using transcriptomic and proteomic analysis. Comparing the marker profile, a high *in vitro*-*in vivo* correlation was shown. Carcinoembryonic antigen-related cell adhesion molecule 5 (CEACAM5), muscarinic acetylcholine receptor M3 (CHRM3), Na^+^-K^+^-Cl^-^ cotransporter 1 (NKCC1), calcium-activated chloride channel anoctamin-1 (ANO1/TMEM16A), and aquaporin-5 (AQP5) are found at significant expression levels in the 3D model. Moreover, cholinergic stimulation with acetylcholine or pilocarpine leads to calcium influx monitored in a calcium flux assay. Cholinergic stimulation cannot be achieved with the sweat gland cell line NCL-SG3 used as a sweat gland model system. Our results show clear benefits of the organotypic 3D sweat gland model versus 2D cultures in terms of the expression of essential eccrine sweat gland key regulators and in the physiological response to stimulation. Taken together, this novel organotypic 3D sweat gland model shows a good *in vitro*-*in vivo* correlation and is an appropriate alternative for screening of potential bioactives regulating the sweat mechanism.

## Introduction

Eccrine sweat glands have the major function in regulating human body temperature via evaporation of sweat [1]. Besides that, they are involved in skin homeostasis, skin hydration and immune defense by secreting moisturizing factors such as lactate and urea [2] and several antimicrobial peptides including dermcidin and lactoferrin [3–6]. Furthermore, sweat glands (SG) harbor stem cell populations [7–9] and have the capacity to regenerate the epidermis during skin wound healing [10]. An average human skin has approximately 1.6–5 million eccrine sweat glands distributed over the whole-body surface with high numbers in palmar, plantar and axillary body regions [11]. Morphologically, they are single tubular structured (exocrine) glands located in the dermis and opening onto the skin surface [2, 12, 13].

The SG consists of a secretory coil (sc), producing isotonic primary fluid predominantly after cholinergic stimulation, next to purinergic or αβ-adrenergic activation [12, 14, 15], and a duct (du), which mostly reabsorbs sodium and chloride ions preventing nutrient loss [16–18]. The secretory coil comprises three different cell types—myoepithelial, secretory clear (serous) and dark (mucous) cells—located within the dermis [19]. The connecting duct has two epithelial cell layers of cuboidal cells—the luminal and basal duct cells—and opens directly onto the skin surface. Each functional cell type expresses several ion channels and specific markers respectively [20], which could display novel targets for the treatment of disorders like hyperhidrosis or for achieving cosmetic effects like reducing unwanted sweat perspiration.

The only cell line derived from the human SG, named NCL-SG3, is commonly used as a 2D model system [21–26]. However, the cells lost their physiological function of cholinergic stimulation by the neurotransmitter acetylcholine (ACh) or the agonist carbachol [22, 27]. *In vivo* muscarinic receptors, mainly CHRM3, are triggered in the secretory coil by cholinergic mediators resulting in sweat generation [11, 28]. Hence, induced cholinergic response is the major route of physiological sweat secretion *in vivo* [20], which is not occurring in the SG cell line NCL-SG3.

This insight implies that additional, more sophisticated *in vitro* 3D cell culture models are needed to allow more comprehensive investigation of the sweat secretion process. Spheroid generation using the hanging drop cultivation technology reflects an optimized method for microtissue modeling [29, 30], so we developed a novel 3D sweat gland model applying this technique. In this study, we show the characterization of primary eccrine SG cells both in 2D culture and in our novel organotypic 3D SG model by analyzing specific markers using gene and protein expression analysis. To provide proof of physiological functionality, 3D models from both axillary and facial SG cells are stimulated and inhibited with agonists and antagonists to show natural, physiological responsiveness in the calcium flux assay. Furthermore, we demonstrate differences in cholinergic response after the inhibition with antagonist glycopyrrolate in 3D models. Thus, this organotypic 3D sweat gland model shall be implemented for the reconstruction of molecular mechanisms of sweat formation and for bioactive or drug screening.

## Materials and methods

### Ethics statement

All experiments were performed in strict accordance with the Declaration of Helsinki. Surgical waste as a donation for sampling does not require any prior authorization by an ethics committee in compliance to regional and national German regulations “Gesetz über die Spende, Entnahme und Übertragung von Organen und Geweben” (§8 ff.). The study was consulted through the plastic surgeons. Biopsies as excess material were obtained with informed written consent from healthy volunteering adults undergoing standard upper arm reduction or facelift cosmetic surgery. The data was analyzed anonymously and only the region of sampling, sex and age of the donor were known.

### Cell culture

#### Sweat gland isolation and cultivation of primary sweat gland cells

Skin biopsies were minced into 1 cm^2^ pieces and digested with 0.5% (w/v) collagenase type V (Merck, Darmstadt, Germany) and 0.25 mg/ml thermolysin (Sigma-Aldrich, Taufkirchen, Germany) in Dulbecco’s modified Eagle’s medium (DMEM, Gibco, Darmstadt, Germany) for 3–5 hours (h) at 37°C in 5% CO_2_. After enzymatic treatment, intact sweat glands were released from the digested skin and transferred with a capillary micropipette to cell culture flasks or to Nunc Lab Tek II chamber slides (#154917, Sigma-Aldrich), which were all coated with Collagen I (BD Biosciences, Heidelberg, Germany). Sweat glands were cultivated in DMEM media supplemented with 30% Ham’s F12 Nutrient Mixture (Sigma-Aldrich), 10% fetal clone serum II (Invitrogen, Carlsbad, CA, USA), 10 ng/ml epidermal growth factor (EGF, Sigma-Aldrich), 100 UI/ml penicillin (Sigma-Aldrich) and 25 μg/ml gentamicin (Sigma-Aldrich) at 37°C in a humidified atmosphere of 5% CO_2_. EGF is notably required for the *in vitro* growth of sweat gland cells like myoepithelial cells as shown in previous studies [31, 32]. The media was changed every 2–3 days and the outgrowing cells were harvested and subcultivated for following experiments.

#### Spheroid generation

Primary subcultivated SG cells are plated in hanging drop 96-well system GravityPLUS (Insphero, Schlieren, Switzerland) in 50 μl containing 10.000–50.000 cells/drop for up to 7 days at 37°C in a humidified atmosphere of 5% CO_2_. Spheroids were harvested in a GravityTrap plate (Insphero) pipetting 100 μl to the top inlet of the plate.

### Histological staining of paraffin sections

Fixation, paraffin sectioning and histological staining were carried out as previously published [33] for an *in vitro* 3D skin equivalent. So, skin tissue and spheroid 3D models were fixed overnight with 4% paraformaldehyde (PFA, Merck, Germany), dehydrated in a graded series of ascending ethanol concentration, cleared in xylene, and embedded in liquid paraffin. Afterwards, cutting of samples into 5 μm-thick sections was performed. Sections were dewaxed in xylene, rehydrated by descendent ethanol concentrations, rinsed with distilled water, stained with hematoxylin and eosin (H&E) or with azan (Heidenhain’s azocarmine and aniline blue), and mounted for histological analysis.

### Cell viability assay

Live-Dead Cell Viability Cytotoxicity Assay (#L3224, Invitrogen) was used to determine live and dead cells in the 3D sweat gland model measuring parameters of cell viability depending on intracellular esterase activity and plasma membrane integrity. Cell culture conditions can negatively affect cell viability by directly or indirectly inducing cytotoxicity, apoptosis, and/or necrosis. The two-color fluorescence kit was performed as whole-mount staining of the 3D SG models per manufacturer’s instructions with 2 μM calcein AM and 4 μm ethidium homodimer-1 (EthD-1) incubated simultaneously for 1 h at room temperature (RT) in the dark. Analysis of labeled cells was directly done after incubation under a fluorescence microscope (Olympus, Hamburg, Germany) using filters for calcein (excitation 488 nm, emission 514 nm) and EthD-1 (excitation 514 nm, emission 633 nm). Quantification of green-fluorescent dye and red-fluorescent labeling was performed using ImageJ software and calculated as corrected total cell fluorescence (CTCF) with CTCF = Integrated Density–(Area of selected cell x Mean fluorescence of background readings).

### Protein expression analysis

#### Immunofluorescence staining

In immunofluorescence staining, a) paraffin sections of human axillary skin tissue, b) adherent *in situ* sweat glands with monolayers of outgrown cells, c) paraffin sections of 3D SG models, and d) whole-mount stained 3D SG models were observed.

Paraffin sections of skin tissue (a) as positive controls and sections of 3D models (c) were deparaffinized, rehydrated by descendent alcohol concentrations, and “antigen-unmasked” by cooking at 95°C in 0.01 M sodium citrate buffer (0.05% Tween 20, pH 6.0) for 15 minutes (min). In the next step, sections were blocked with 10% normal goat serum (Sigma-Aldrich) diluted in phosphate buffered saline (PBS, Gibco) containing 0.1% Triton X-100 for 1 h at RT. For protein expression analysis in adherent *in situ* glands with outgrown monolayer SG cells (b) and for whole-mount staining of 3D SG models (d), samples were fixed in 4% PFA, blocked and permeabilized in the manner described above.

Next, primary antibodies against CEACAM5 (1:500 dilution, rabbit polyclonal, #Ab15987, Abcam, Cambridge, United Kingdom), ANO1 (1:100, mouse monoclonal, #Ab190721, Abcam) and α-SMA (1:500, mouse monoclonal, #M0851, Dako, Glostrup, Denmark) diluted in Dako Antibody Diluent with background reducing components (#S3022, Dako) were incubated in a humid chamber at 4°C overnight in the dark. Afterwards, samples were washed 3 times in PBS, incubated with the corresponding secondary antibodies (1:200, Alexa Fluor 488 rabbit anti-mouse IgG (H+L) or Alexa Fluor 488 goat anti-rabbit IgG (H+L), Invitrogen) and with 1 μg/ml 4’, 6-diamidino-2-phenylindole (DAPI, Sigma-Aldrich) counterstaining cell nuclei for 1 h at RT diluted in the same Dako antibody solvent as described above. Negative controls were incubated just with the corresponding secondary antibody to determine non-specific staining (data not shown). Rinsing steps were all performed with PBS. Finally, samples were mounted with Dako Fluorescence Mounting Medium (Dako, Denmark) and microscopy was performed by using a fluorescence microscope (Olympus, Hamburg, Germany) or a confocal laser scanning microscope (CLSM, Leica DMi8, Wetzlar, Germany) with appropriate filters. For confocal imaging analysis, whole mount-stained 3D SG models were analyzed with “LAS X” Leica software using Z-stacking and scanned every 5 μm.

#### Protein analysis via Western blotting

For Western blotting, 2D and 3D cell culture samples were pelleted and lysed in 50 mM Tris-HCl buffer with protease inhibitor cocktail (Sigma-Aldrich). 10 μg of cleared lysate of protein samples were resuspended in 2x Laemmli sample buffer (4% SDS, 10% mercaptoethanol, 20% glycerol, 0.004% bromophenol blue, 0.125 mM Tris-HCl, pH 6.8). Samples were denatured at 95°C for 10 min. Supernatants were subjected to SDS page gel electrophoresis for approximately 60 min at 200 V in running buffer (25 mM Tris base, 190 mM glycine, 0.1% SDS, pH 8.3) using 4–12% gradient gels (Invitrogen). After transferring the proteins to a nitrocellulose membrane (Invitrogen) at 170 mA for 2 h and after ponceau S staining, membranes were blocked with 3% non-fat milk in TBST (20 mM Tris, 150 mM NaCl, 0.05% Tween-20, pH 7.4) for 60 min at RT. Incubation with primary antibodies (anti-AQP5, 1:500, rabbit monoclonal, #Ab92320, Abcam; anti-GAPDH, mouse monoclonal, 1:1000, #ACR001P, Acris Antibodies, Herford, Germany) diluted in TBST containing 5% non-fat milk at 4°C overnight, 3x washing of membranes in TBST, and incubation of HRP-conjugated secondary antibody (1:2500 diluted in the same antibody solvent, goat anti-Mouse IgG (H+L), #AP124 or goat anti-Rabbit IgG (H+L), #AP132, both Merck Millipore, Darmstadt, Germany) was performed for 1 h at RT. Next, membranes were 3x washed in TBST and incubated with Amersham ECL Western Blotting Detection Reagent (#RPN2106, GE Healthcare, Little Chalfont, United Kingdom) for 2 min at RT. Detection and quantitative analysis was performed with TotalLab Quant Software and observed pixels were normalized to GAPDH loading control samples.

### RNA isolation and expression profiling via qRT-PCR

Total RNA from isolated sweat glands, 2D, and 3D cell culture samples was extracted by a conventional method according to manufacturer’s instructions (RNeasy Micro Kit, #74004, Qiagen, Hilden, Germany). cDNA was synthesized in reverse transcription using 500 ng total RNA and random primers (Invitrogen) carried out as previously published [33]. qRT-PCR was performed with sense and antisense oligonucleotides by 40 amplification cycles (melting step at 95°C for 30 sec, annealing step at 58°C for 60 sec, and amplification step at 72°C for 30 sec). Primer sets: CHRM3: forward: 5’-ACAATAAGGTTTTGCTGTGG-3’ and reverse: 5’-AACCAATACAATGTGTCCAG-3’; AQP5: forward: 5’-CCACCTTGTCGGAATCTACTT-3’ and reverse: 5’-CTGAACCGATTCATGACCAC-3’; ANO1: purchased from Qiagen (#QT00076013); NKCC1: forward: 5’-AGGATGGCAAGACTGCAACT-3’ and reverse: 5’-CGTGCAACTGGGAGACTCAT-3’; CEACAM5: forward: 5’-TCATCCTGAATGTCCTCTATG-3’ and reverse: 5’-GTGATGTTGGAGATAAAGAGC-3’; G6PDH: forward: 5’-ATCGACCACTACCTGGGCAA-3’ and reverse: 5’-TTCTGCATCACGTCCCGGA-3’ as the housekeeping gene. Fold difference of gene expression was determined by the ΔΔCT method.

### Calcium flux assay with fluo-4

Calcium signaling was analyzed in 3D SG models cultured with primary facial or axillary SG cells. Experiments were performed in 96-well black-walled, clear-bottom microplates (#CLS3340, Sigma-Aldrich) and in indicator-free DMEM media (#31053, Gibco) at RT. Cells were incubated with 4 μM fluo-4 acetoxy-methyl (AM) ester (#F14217, Invitrogen) for 1 h after washing the cultures twice with PBS. De-esterification was done for 1–2 h to allow cleavage of the fluorophores by intracellular esterases, following the manufacturer’s protocol. 2,5 mM Probenecid (#P8761, Sigma-Aldrich) was added to the media preventing the efflux of the calcium indicator after loading. After baseline recording (excitation 485 nm, emission 535 nm), cells were stimulated with agonists ACh, pilocarpine, carbachol, adenosine triphosphate (ATP), and/or inhibited with antagonists glycopyrrolate (all Sigma-Aldrich). Relative change for intracellular calcium fluxes was calculated by equation ΔF = (F_max_−F0)/F0 in at least three independent experiments with n = 6±SEM.

### Statistical analysis

Data represent mean values and error bars display standard error of the mean (SEM). Analysis of statistical significance was calculated with GraphPad Prism 5.0 software by Student’s t-test, Mann-Whitney U test for nonparametric data or One-way ANOVA (analysis of variance) comparing means of three or more samples, followed by Bonferroni post-hoc test (α = 0.05). Differences were considered statistically significant with p<0.05(*), p<0.01(**) and p<0.001(***).

## Results

In this study, we investigated the morphological differences and changes in gene and protein expression of specific eccrine SG markers in our novel organotypic 3D SG model, primary 2D cultures and native human sweat glands. Furthermore, our study elucidated the organization and the differentiation of primary SG cells in the 3D model. The establishment and orientation of an apical-basal polarity axis was analyzed *in vitro* and major functions of the eccrine sweat gland like cholinergic stimulation could be shown.

### Morphological and histochemical analysis of the organotypic 3D SG model

**Fig 1:**
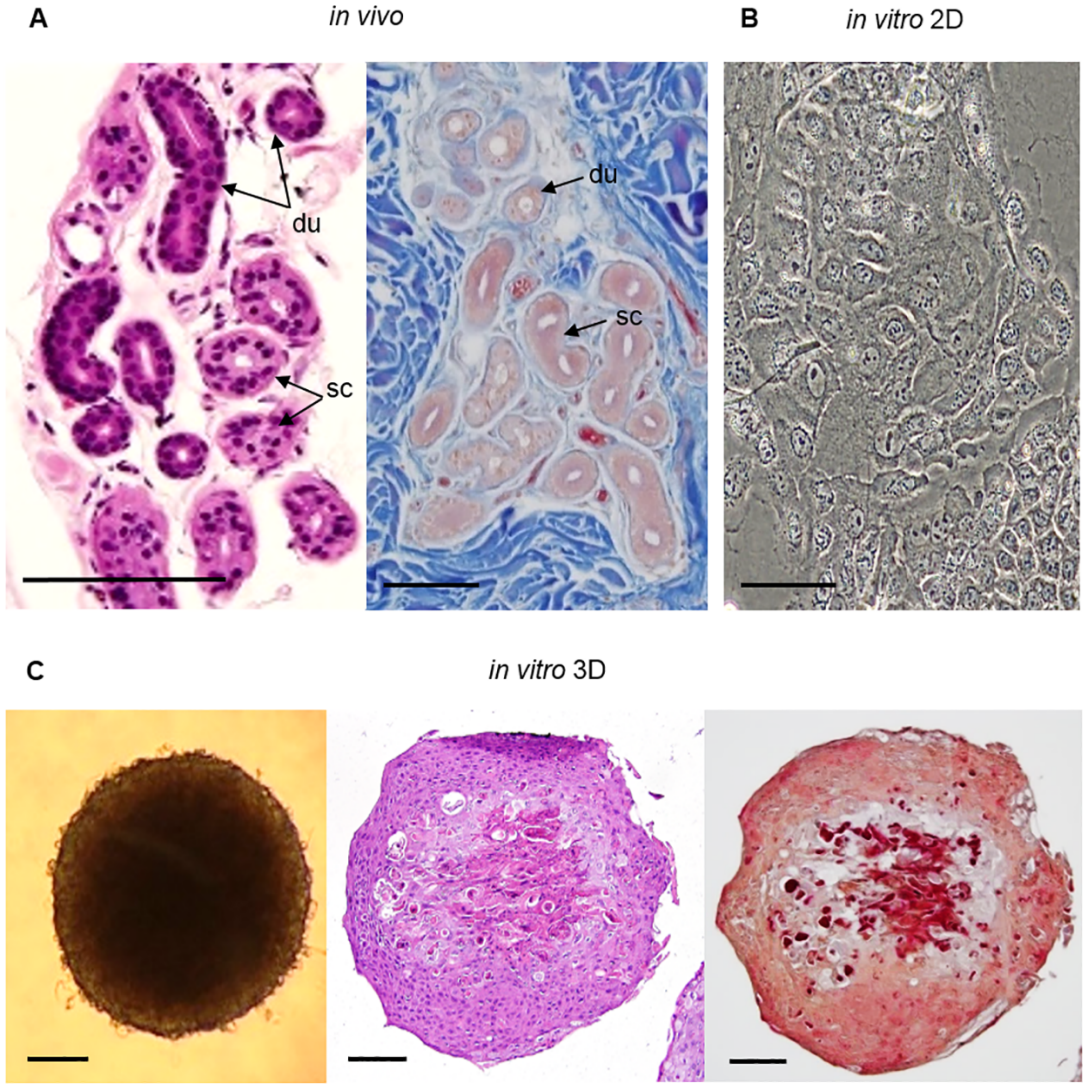
Histological analysis of human eccrine sweat glands, monolayer cells and 3D cell culture models *in vitro*. (A) H&E and azan staining of paraffin axillary skin tissue sections visualizing the morphology of native eccrine SG’s with secretory coil portions (sc) and ductal portions (du) of the gland. (B) Bright-field microscopy of primary SG cells in 2D culture with their typical ‘cobblestone’ morphology is shown. (C) Bright-field microscopy and H&E-/azan-stained sections of a differentiated SG 3D model with irregularly arranged, multilayered cells and less dense cells in the center of the spheroid. Collagen in blue could not be detected in azan staining, but acidic structures like nuclei or matrix were stained in red or dark red. Scale bars 100 μm.

We first analyzed the morphology of the spheroid-shaped organotypic 3D SG model and compared it to the *in vivo* situation of native human SG’s. The morphological and histochemical analysis of this novel scaffold-free 3D SG model was determined in bright-field microscopy, by hematoxylin and eosin staining (H&E), and azan staining of paraffin sections of skin tissue and of the 3D SG model (Fig 1). In H&E-stained sections of native SG’s, ductal portions (du) were distinguished from secretory coil portions (sc) by their intensive purple double-layered epithelium and their luminal stratified border (Fig 1A). Primary eccrine SG cells *in vitro* showed the characteristic ‘cobblestone’ morphology (Fig 1B) [34]. H&E- and azan-stained paraffin sections of the novel 3D SG model revealed a differentiated and segmented structure of primary eccrine SG cells in the spheroids (Fig 1C). The cells of the model were arranged irregularly and the multilayered cells were distinguished by different colors in H&E- and azan-staining. The H&E- and the azan-stained sections of the sphere showed less dense organized cell nuclei with partly unstained fractions in the center, but unlike the *in vivo* sweat gland with an unstained hollow lumen in the coil and in the duct portion. The presence of matrix was possibly implied by the dark red staining of the center previously demonstrated by Diekmann et al. in the subcutaneous part of *in vitro* 3D skin models [33]. In an *in vitro* 3D model, orientation and positioning is controlled by the apical-basal axis, which determines the tubular tissue architecture [35]. The responsible relationships for differentiation of epithelia during 3D reconstruction like the interaction of cells with extracellular matrix (ECM) and the surrounding media [36] could indicate these regulatory processes leading to apical-basal tissue architecture in our 3D SG model. Further research is required to provide evidence of matrix existence and of distinct tissue types.

### Cell culture optimization of 3D SG models regarding viability

**Fig 2:**
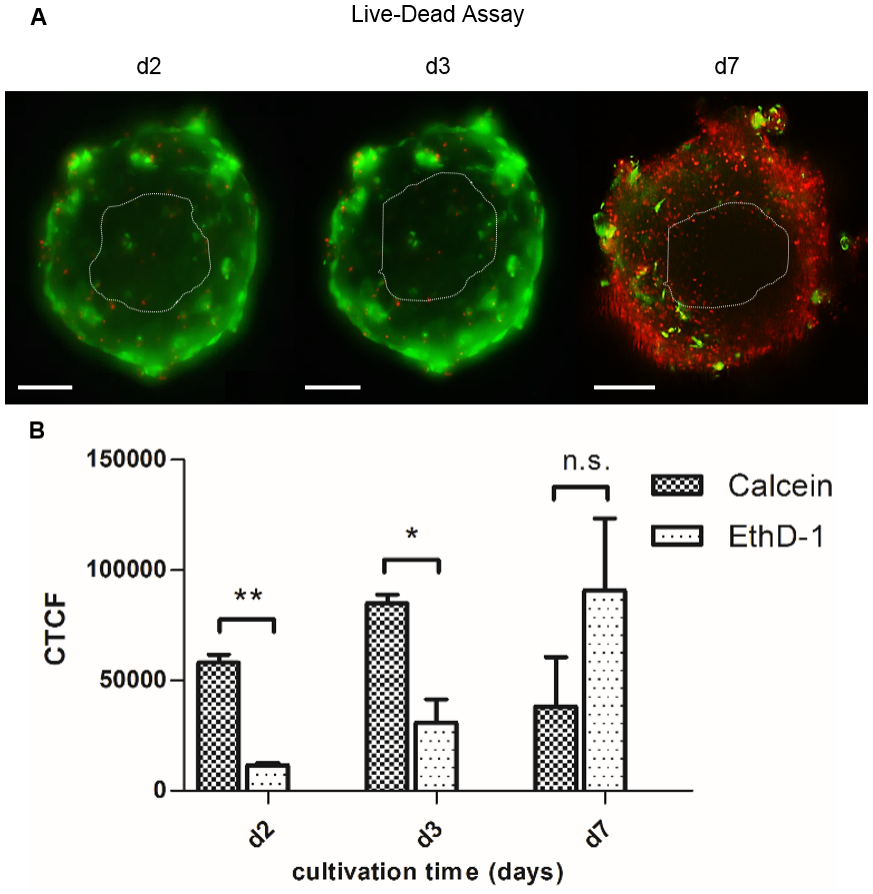
Viability of sweat gland cells in 3D hanging drop cultures. (A) By Live-Dead Assay, viability of 3D models cultured for 2–7 days (d2, d3, and d7) was examined via whole-mount staining of spheroids with green-fluorescent dye calcein and red-fluorescent dye EthD-1. (B) Quantification of green fluorescent marker and red labeling of dead cells as corrected total cell fluorescence (CTCF) in C (Student’s t-test with n = 3; mean±SEM; p<0,05(*)<0,01(**); n.s.: no significance). Optimum cultivation time with d2-d3 with significant level of viable cells compared to dead cells. Scale bars 100 μm.

Duration of culturing is a crucial part in the optimization process of cell tissue cultures, since e.g. hypoxia or other chemical gradients in spheroids can negatively influence cell conditions [37]. To define the optimal cultivation time, we gained insights into the viability of the cells cultured in 3D in dependence on the culture time (Fig 2A). We verified this by quantification of calcein staining assigned viable cells and EthD-1 labeled dead cells in the Live-Dead Assay (Fig 2B). The data indicate a cultivation time of 2–3 days as optimal culture time for the 3D SG model with significant differences between viability and cell death. At the later time points d3 to d7, the viability decreased regarding the corrected total cell fluorescence (CTCF) of calcein-stained cells. CTCF of EthD-1 staining representing dying and dead cells was increased 2.4-fold in hanging drops with extending the cultivation time to d7.

### Gene expression of primary SG cells cultured in 2D versus 3D

**Fig 3:**
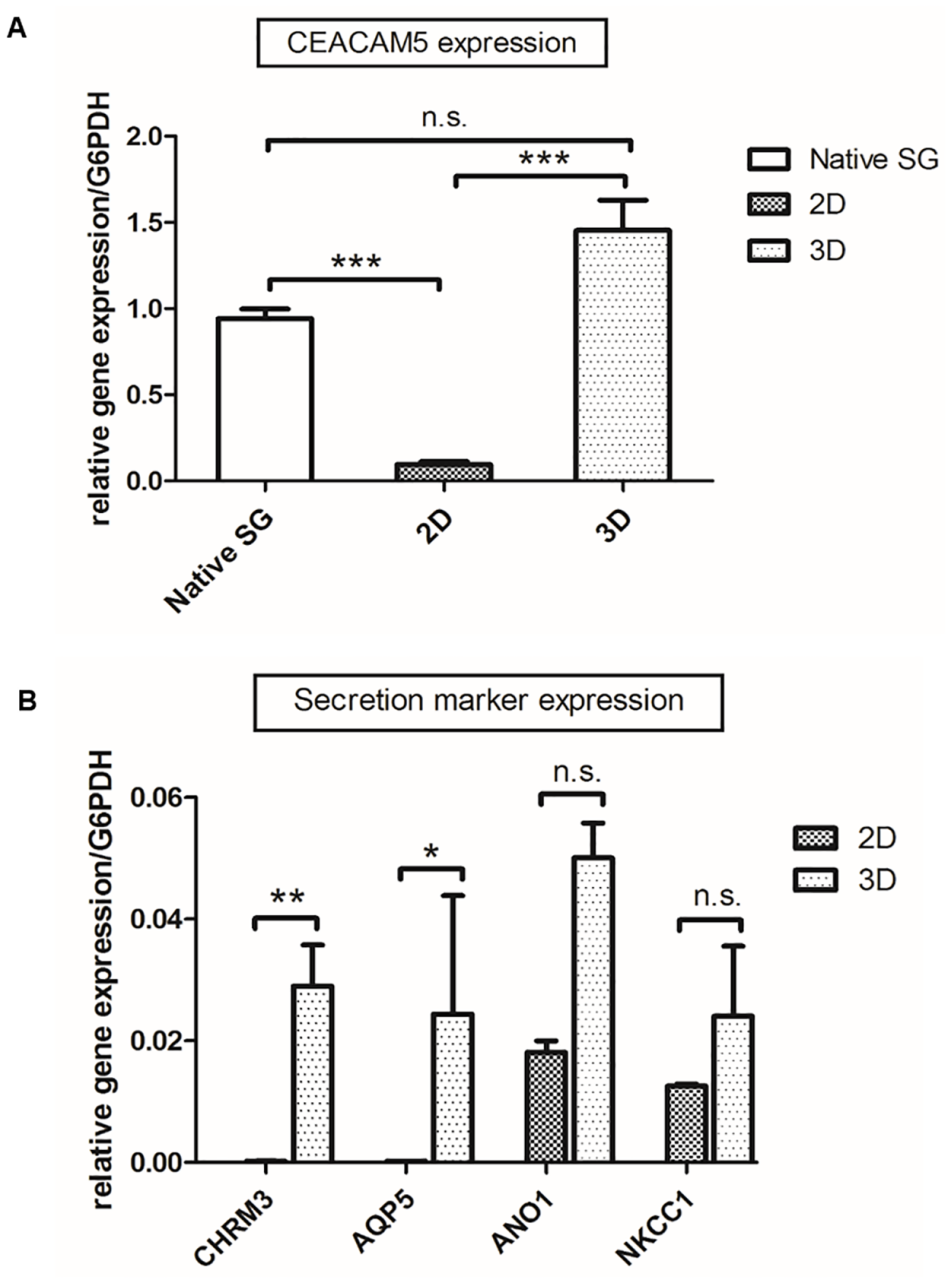
Gene expression profile analysis via qRT-PCR of *in vitro* cultures of the eccrine sweat gland. (A) In multicellular spheroids, expression of CEACAM5 as a differentiation-related marker was significantly induced compared to 2D cultures. Further, gene expression in 3D was on a similar level to native sweat glands (One way ANOVA; n≥8, mean±SEM, p<0,05(*)<0,01(**)<0,001(***) with Bonferroni Post-hoc test). (B) Significantly increased gene expression levels of the specific secretion marker genes CHRM3 and AQP5 could be obtained by cultivation of SG cells in 3D models (Student’s t-test; n≥10, mean±SEM, p<0,05(*)<0,01(**)<0,001(***)). NKCC1 and ANO1 expression was improved in 3D spheroids, but not significantly. G6PDH was used as housekeeping gene.

We elucidated the expression of specific human eccrine sweat gland markers in 2D and 3D cultures by qRT-PCR (Fig 3). CEACAM5 as a differentiation and polarity marker [38] is expressed at similar levels in both 3D SG models and native SG. In primary 2D cultures, CEACAM5 expression is significantly reduced (Fig 3A). Next, expression of CHRM3 as a receptor transmitting cholinergic trigger and further crucial markers mediating sweat secretion including the sodium-potassium-chloride cotransporter NKCC1, luminal chloride channel ANO1, and the water channel AQP5 were analyzed in primary 2D cultures and in the 3D SG model (Fig 3B). Here, we observed significantly reduced expression levels of CHRM3 and AQP5 in 2D cultures as compared to the 3D SG model. In contrast, no significant differences were found for ANO1 and NKCC1 gene expression in 2D cultures versus 3D models.

### Apical-basal orientation in 3D SG models

Immunofluorescence analysis was performed to gain insight into the localization of relevant marker proteins and into the apical-basal orientation of eccrine sweat gland cells cultivated in the organotypic 3D SG model.

**Fig 4:**
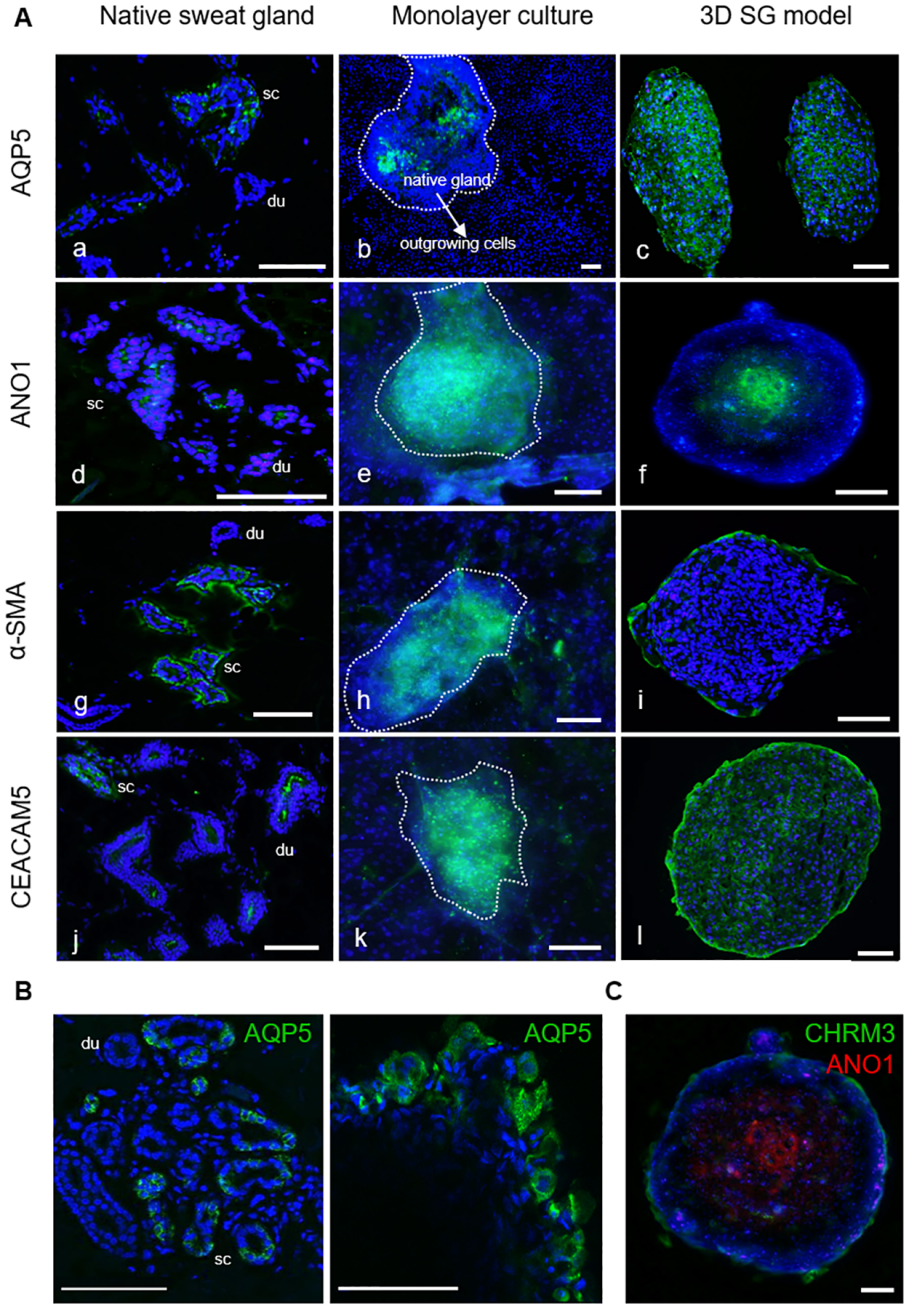
*In vitro* characterization of key marker proteins AQP5, ANO1, α-SMA, and CEACAM5 in cultures of the SG by immunofluorescence staining. (A) Stained secretory coil (sc) and duct (du) sections of native human sweat glands were analyzed in axillary skin tissue (a,d,g,j) compared to *in situ* glands (dashed lines) with outgrowing cells in 2D cultures (b,e,h,k) and 3D models (c,f,i,l). Polarization and differentiation of spheroids could be shown with luminal ANO1 (f) and basolateral α-SMA staining (i). Outgrown SG cells showed decreased marker expressions in the 2D cultures. Nuclei stained with DAPI. (B) By confocal imaging, the localization of AQP5 was investigated in sections of native skin tissue with eccrine sweat glands and in whole mount-stained 3D models. *In vivo* and *in vitro*, AQP5 could be shown in the cytoplasm, in the basolateral and the luminal membrane of secretory coil cells and in cells of the 3D model. (C) Localization of ANO1 and CHRM3 was analyzed to investigate the apical-basal polarity in the 3D model. Here, the whole mount-stained 3D model indicated this histoarchitecture with luminal ANO1-staining in the center and basolateral CHRM3-staining in the outer layer of the sphere. Scale bars 100 μm.

In histological sections of native skin tissue, the water channel AQP5 was present in secretory cells and not in the ductal portion (Fig 4A+4B). AQP5 protein expression could only be determined in *ex vivo* adherent glands, not in 2D outgrown cells (Fig 4Ab). In sections of the novel 3D model, AQP5 was strongly localized intracellularly (Fig 4Ac). Furthermore, ANO1 expression was studied as a specific marker for the apical orientation of the cell. This Ca^2+^-activated chloride channel was detected in the luminal part of the secretory coil *in vivo* as well as in luminal cells *in vitro* in the 3D SG model (Fig 4Ad+4Af). For the distinction of myoepithelial cells possessing stem cell potential [39], α-SMA staining was investigated labeling cells encompassing the spheroid as an outer cell layer (Fig 4Ag–4Ai). In sections of human skin tissue, α-SMA was strongly localized in surrounding myoepithelial cells of the secretory domain and diminished in the ducts. α-SMA expression was only determined in a few 2D outgrown cells, in contrast, to cells of the 3D model, where α-SMA labeling was most prominent in the basolateral cell layer comparable to *in vivo* (Fig 4Ai). The expression of CEACAM5 as a prominent marker of eccrine sweat gland cells [10, 38] was also analyzed (Fig 4C). The glycoprotein CEACAM5 plays a key role in protein sorting, secretory mechanisms and cell adhesion. Furthermore, CEACAM5 is associated to functions like cell surface recognition and proper organization of surface structures [38]. Immunofluorescence staining of CEACAM5 indicated a strong luminal staining in native ducts and a positive CEACAM5 expression in cells of the secretory coil *in vivo* (Fig 4Aj). In the *ex vivo* gland and in outgrown monolayer cells, CEACAM5 was strongly determined in the adherent gland and less expressed in outgrown, proliferating cells (Fig 4Ak). CEACAM5 was highly distributed in the 3D model, but also with a strong staining in the outer membrane of the sphere (Fig 4Al).

To further study the epithelial polarity, the expression of the marker protein AQP5 was analyzed in a higher magnification by confocal imaging (Fig 4B). Localization of AQP5 was investigated in native skin tissue sections with eccrine sweat glands, compared to the whole mount-stained 3D SG model. *In vivo*, AQP5 was detected in the secretory coil—but not in the duct section—as well as in cells of the 3D SG model *in vitro*. AQP5 was determined in the cytoplasm and in the overall surrounding membrane of these cells, apical and basolateral in the same manner.

In a further study, the organotypic, epithelial apical-basal polarity of the 3D model was analyzed by co-staining of the luminal marker ANO1 and the basolateral marker CHRM3 in the 3D model (Fig 4C). Here, the whole mount-stained 3D model indicated this histoarchitecture with luminal ANO1-staining in the center and basolateral CHRM3-staining in the outer layer of the sphere.

In total, we have shown that relevant functional sweat gland proteins were lost during cultivation in 2D. Further, the data show the differentiation and the polarity of cells cultured in the organotypic 3D model as well as the expression of key sweat gland proteins being involved in the secretory process.

### Quantification of AQP5 as a secretion marker in 2D and 3D cultures

Sweat mainly consists of sodium chloride (NaCl) and water. The function of AQP5 has not been full characterized [40], but after translocation to the apical membrane this channel allows water entering the lumen of the coil [41]. This indicates the contribution of AQP5 to the sweat secretion mechanism [40, 42].

**Fig 5:**
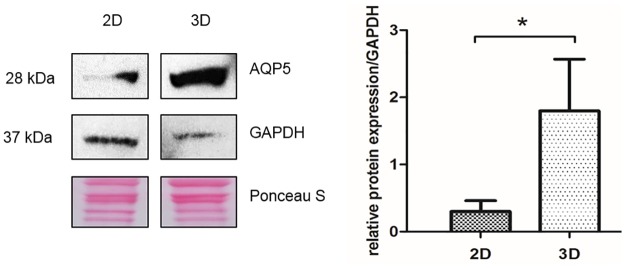
Induced AQP5 protein expression in 3D derived sweat gland cells. Quantification of Western blot signals relative to GAPDH, shows strong increase in total AQP5 protein expression (28 kDa) as a secretion marker of clear cells in 3D hanging drop models. Loading controls GAPDH (37 kDa) and Ponceau S staining. Significance calculated with Student‘s t-test (n = 3; mean±SEM) with p<0,05(*).

Cultivation of primary SG cells in traditional 2D cultures showed loss of functions for key proteins like AQP5. Different to our previous immunofluorescence staining of 2D cultures with diminished AQP5 expression (Fig 4Ab), we observed only weak protein expression of AQP5 in primary SG cells cultured in 2D by using western blotting. In contrast, the total amount of AQP5 is significantly increased in 3D SG models not directly indicating functionality (Fig 5). Immunofluorescence staining and western blot analysis approved evidence of reduced AQP5 expression in 2D cultures, and thus showed the advantages of our organotypic 3D SG model.

### Physiological functionality of sweat gland cells *in vitro*

**Fig 6:**
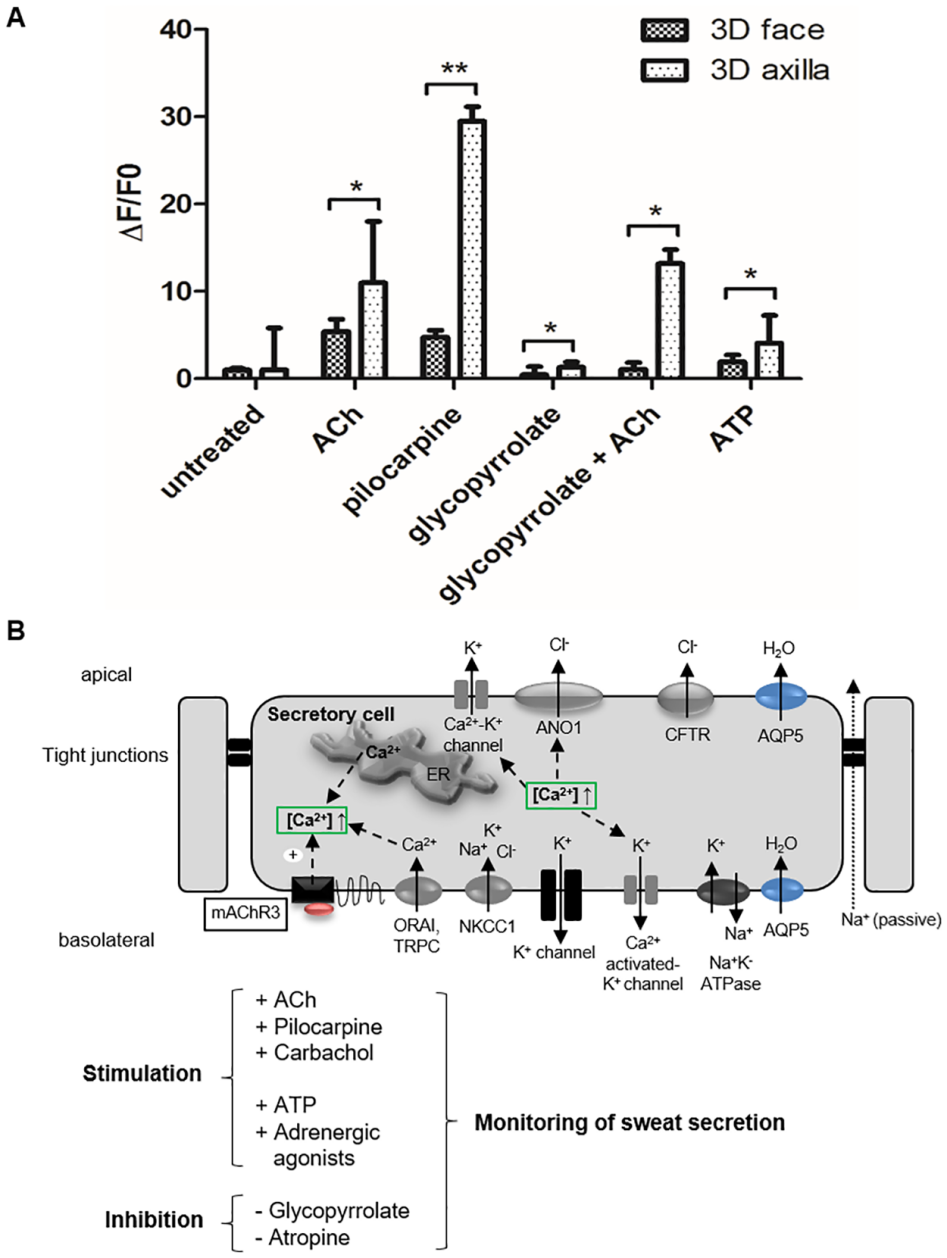
Stimulation and inhibition of sweat gland cells monitored with calcium indicator fluo-4. (A) Fluorescence levels (ΔF/F0) in 3D SG models derived from facial or axillary sweat glands show stimulation and inhibition of receptors/ion channels in 3D SG models with fluo-4 (Student’s t-test with n = 6; mean±SEM; p<0,05(*)<0,01(**)). Muscarinic receptor (mAChR) agonists (ACh, pilocarpine) and purinergic stimulation (ATP) increased intracellular Ca^2+^-levels, but remarkably significant in axillary 3D SG models. Muscarinic antagonist, glycopyrrolate, inhibits stimulation with ACh only in axillary 3D SG models. (B) Schema of key receptors and target ion channels in secretory clear cells involved in sweat secretion. Ca^2+^ influx mediated by channels in endoplasmic reticulum (ER) and by ORAI/TRPC channels in plasma membrane.

To assess physiological functionality of this novel *in vitro* 3D SG model, intracellular calcium mobilization was investigated in the calcium flux assay. *In vivo*, sweat secretion is triggered by the stimulation of muscarinic and purinergic receptors in response to specific mediators like ACh. Stimulation leads to influx of the ubiquitous second messenger calcium from intracellular calcium stores and from the interstitial fluid into the cell cytoplasm increasing fluo-4 fluorescence intensity. In our calcium flux assay, the stimulation capacity of sweat gland cells originated from different body regions was elucidated (Fig 6). The analysis was performed with the organotypic 3D SG models cultured either with facial or with axillary eccrine sweat gland cells.

Treatment with the cholinergic agonists acetylcholine and pilocarpine resulted in increased Ca^2+^ levels in both 3D SG models (Fig 6A). However, we were surprised to find significant differences in the cholinergic response of both 3D models originating from axilla and face cells: a 2.0-fold increase of Ca^2+^ levels in ACh-stimulated and a 6.2-fold increase in pilocarpine-stimulated axillary 3D SG models could be observed as compared to equally treated facial 3D SG models. Muscarinic receptor antagonist glycopyrrolate functions as a competitive, anti-cholinergic compound and does not itself influence the intracellular calcium concentration. In combination with ACh, cholinergic stimulation in glycopyrrolate-treated 3D models can be drastically inhibited in SG cells derived from skin facial tissue, but not in cells derived from axillary tissue. Adenosine triphosphate (ATP) indicates stimulation via endogenous purinergic receptors and led to an increase in intracellular calcium causing Ca^2+^-dependent Cl^-^ efflux via ANO1 [23]. Here, 3D SG models respond to natural purinergic stimulation, which is also significantly increased in axillary SG models as compared to facial 3D SG models.

Our findings have been incorporated into a schematic model showing a part of the dynamic and multifaceted sweat secretion process in a secretory clear cell (Fig 6B). This scheme suggests a working model of ion channels as well as receptors, which were triggered in the calcium flux assay, located on the apical and basolateral side of secretory cells from the eccrine sweat gland coil *in vivo*. These represented targets are affected by the applied cholinergic agonists and antagonists and led to changes of intracellular calcium (ΔF/F0) visualized by fluo-4.

Overall, we could show for the first time cholinergic *in vitro* stimulation of primary human eccrine sweat gland cells in our novel organotypic 3D SG model. Surprisingly, significant differences in cholinergic and purinergic stimulation were obtained in the 3D SG models originating from axillary SG cells with strongly increased intracellular calcium as compared to 3D SG models cultured with facial cells. Due to the different levels of stimulation between conventional cell lines and 3D cultures, we postulate a strong advantage using the 3D system reflecting the *in vivo* situation of a functional secretory sweat gland more closely.

## Discussion

Eccrine sweat glands maintain body temperature [1], secrete antimicrobial peptides, contribute to skin homeostasis [2–6] and harbor progenitor stem cells involved in wound healing of the human skin [7–10]. However, this gland is not fully characterized as appropriate and *in vitro* 3D models were lacking, which could enhance research on sweat secretion mechanisms and would be feasible for screening of bioactives or drugs screening against disorders like hyperhidrosis. The results of our study demonstrate the development and the characterization of a novel and functional organotypic 3D model of the human eccrine sweat gland applying the scaffold-free hanging drop cultivation technology. This 3D model revealed strong advantages versus conventional *in vitro* 2D cultures. It shows cellular differentiation resulting in an apical-basal polarity axis, the gene and protein expression of eccrine sweat gland markers related to sweat secretion, and physiological functionality.

First, cell culture conditions were optimized by a viability assay displaying an incubation time of 2–3 days as an optimum for the proliferation and differentiation of these 3D SG models. This finding is further supported by Bartosh et al., who reported a similar optimal time frame for the aggregation and cultivation of multipotent mesenchymal stromal cells using the hanging drop technique [29]. Nonetheless, this promising 3D model requires further investigation due to its limitations in viability to warrant long-term cultivation. It was suggested that spheroid cultures possess chemical gradients (oxygen, nutrients or catabolites) leading to suboptimal culture conditions, quiescence and apoptosis. Furthermore, it is discussed that these quiescent and apoptotic cells migrate to the center of the spheroid [37]. However, it remains open whether the histoarchitecture of the sphere is processed by apoptosis and/or necrosis or by apical-basal axis orientation of the cells in the 3D SG model indicated by immunofluorescence staining (Fig 4C).

Second, the epithelial apical-basal polarity within the 3D model was shown by histochemical analysis with specific SG cell marker proteins like AQP5, CHRM3, CEACAM5, and ANO1. Li et al. demonstrated a comparable reconstruction of sweat gland-like structures with Matrigel-cultivated primary cells, which only formed tubular-like structures after transplantation in mice [36, 43]. However, the transplantation step makes their model more complicated as compared to our hanging-drop culture and would not be suitable for applications, which prohibit the use of animal models such as screening for cosmetic bioactives. The cellular polarization in our 3D SG model was investigated via co-localizing of ANO1 as an apical marker [23] and CHRM3 as a basolateral marker [20]. In this immunofluorescence staining, apical-basal polarity could be demonstrated in a whole mount-stained 3D model (Fig 4C). Already with the current results of gene expression analysis the superiority of the organotypic 3D SG model versus conventional 2D cultures is clearly supported since significant expression levels of specific eccrine sweat gland markers like CEACAM5, CHRM3, and AQP5 were detected to a higher value (Fig 3).

In addition, AQP5 protein synthesis was significantly increased in 3D models, demonstrating a functionally required marker for sweat secretion [41]. AQP5 is dynamically regulated by multiple mechanisms described by Kitchen et al.: (i) at transcriptional/translational level; (ii) by conformational change or “gating” and (iii) by translocation to the membrane in response to a trigger [44]. In the unstimulated state of the cell, Nejsum et al. and Du et al. could show AQP5 abundant in the apical and in the basolateral membrane of secretory cells [41, 45]. Here, AQP5 was also not specifically connected to the apical membrane of unstimulated secretory coil cells *in vivo* and *in vitro*. During secretion AQP5 is translocated to the apical membrane of secretory clear cells allowing water to enter the lumen of the coil [40, 42]. Further, Ishikawa et al. described the translocation of AQP5 in parotid glands after cholinergic CHRM3 stimulation and could show a quick converse translocation from the apical membrane to the cytoplasm [46]. This raises the question if this mechanism of AQP5-translocation could be demonstrated in cells of our 3D model. Although the function of this water channel is not fully characterized, increased distribution of AQP5 was previously reported in hyperhidrotic sweat glands typified by uncontrolled and excessive sweating [40]. In our 3D SG model AQP5 expression was significantly induced (Figs 3–5). It is tempting to speculate that the total amount of AQP5 in our cultures affects fluid production. This may lead to the assumption that higher total protein expression of AQP5 represents an increase in active secreting sweat gland cells in our 3D SG model. However, it remains unclear if increased protein expression of this water channel implicates functionality and “gating” after cholinergic stimulation in eccrine sweat gland cells of this 3D model.

In the third part of the study, we further proved the relevance of our organotypic 3D SG model regarding physiological functionality by evaluating the cell response in the calcium flux assay after cholinergic or purinergic stimulation. Binding of stimulators to their respective receptors activates the calcium signaling pathway, most likely through phospholipase C (PLC), finally triggering sweat secretion [20]. We have proven that primary eccrine sweat gland cells in 3D have the capacity to react to cholinergic mediators, such as ACh and pilocarpine, and purinergic agonists, like ATP, and could be specifically inhibited by the muscarinic antagonist glycopyrrolate (Fig 6A) [20]. Cholinergic or α-adrenergic stimulation of secretory cells of the sweat gland leads to intracellular Ca^2+^-uptake resulting in opening of K^+^ and Cl^-^ channels, thus activating NKCC1 cotransporter [12, 47]. This process *in vivo* results in the formation of hypotonic primary sweat and is thought to involve more still unknown components required for sweat secretion.

Surprisingly, glycopyrrolate, a cholinergic antagonist, reacted differently in the 3D model generated from axillary or facial primary sweat gland cells. Only in the model from facial skin an inhibited Ca^2+^ influx was reported (Fig 6A). In line with these results, cholinergic stimulation with ACh and pilocarpine as well as purinergic stimulation with ATP showed significantly increased intracellular Ca^2+^ levels in axillary 3D SG models. The significant cholinergic differences are not expected to be based on apocrine axillary sweat gland cells contaminating the 3D models. Thus, apocrine sweat glands do not respond to a cholinergic stimulus and possess β-adrenoceptor subtypes as well as purinoceptors [48]. This suggests that apocrine glands are stimulated by catecholamines like adrenergic agonists but not via cholinergic agonists inducing the significance in stimulation. These data raise the question whether axillary sweat glands exhibit stronger cholinergic sensitivity. It will be intriguing to test whether G-protein-coupled receptors like CHRM3 are exposed to higher amounts on the cell plasma membrane in *in vivo* axillary sweat glands as compared to our *in vitro* 3D SG model. In addition, the sweat gland cells of the 3D models originated from different donors. However, Papadimitropoulos et al. reported a decrease in inter-donor variability and a significant upregulation of multipotency-related gene clusters following 3D cultivation [49]. Future research is needed to address these unanswered questions.

In general, 3D cell culture model systems mimic and reconstitute the *in vivo* situation considerably better than 2D cell cultures [30, 34, 36, 50]. The eccrine sweat gland cell line NCL-SG3 is still widely used as a sweat gland model system [21], although it remains unclear from which cell types it is derived and which cell types it contains—clear, dark or myoepithelial cells [23]. Interestingly, cholinergic and α-adrenergic stimulation could not be shown in these cells [22, 24–27, 51], so a loss of function of crucial receptors in signaling pathways could be speculated. In contrast, CHRM3 was highly expressed in our organotypic 3D SG model verified by qRT-PCR (Fig 3) and in physiological response to cholinergic agonists (Fig 6). To our best knowledge, this novel 3D SG model demonstrates for the first time a well-defined and physiological *in vitro* model with properties of the secretory cell subpopulations of a native human eccrine sweat gland. However, additional characterization of cell type-specific regulatory ion channels and transporters contributing to sweat secretion like cystic fibrosis transmembrane conductance regulator (CFTR) [52] should be investigated in further studies.

In addition to the sweat secretion process, sweat glands are also involved in the epidermal regeneration process [10, 53], harbor progenitor stem cell sources in the sweat duct [9] and in the stroma of sweat glands [7, 8]. The intermediate filament protein and stem cell marker Nestin is expressed in 80% of 2D primary sweat gland stroma-derived stem cells *in vitro* [7, 54]. In further studies, the existence of highly proliferating and regenerative stem cells in our 3D model should be addressed, previously shown in Matrigel-cultured 3D models of myoepithelial subpopulation [39]. In line with this approach, our 3D SG models could also be implemented into a full skin model missing skin appendages like sweat glands [33, 55–57] for studying wound healing processes or the multifaceted and dynamic sweat secretion mechanism, which remains incomplete [20, 47, 58]. In addition, fundamental research in this field could replace animal modeling with these functional 3D models as an alternative disease model, e.g. for examining hyperhidrosis, or for pharmaceutical drug and cosmetic bioactive screening.

Taken together, our organotypic 3D SG model not only integrates regulative and major components relevant for sweat secretion, but also displays the physiological regulation of the sweat secretion process making it highly relevant for studying unknown mechanisms of sweating *in vitro*. With our novel organotypic 3D SG model, we can accelerate basic research of both eccrine sweat gland development, molecular and regulative sweat secretion processes, wound healing or drug-screening in disease modeling.
